# Y-STR Databases—Application in Sexual Crimes

**DOI:** 10.3390/genes16050484

**Published:** 2025-04-25

**Authors:** Rita Costa, Jennifer Fadoni, António Amorim, Laura Cainé

**Affiliations:** 1 Faculty of Medicine, Porto University, 4200-319 Porto, Portugal; up201906725@fc.up.pt; 2 National Institute of Legal Medicine and Forensic Sciences, I.P., North Branch, 4050-202 Porto, Portugal; jennifer.n.fadoni@inmlcf.mj.pt (J.F.); antonio.j.amorim@inmlcf.mj.pt (A.A.); 3 LAQV&REQUIMTE, Laboratory of Applied Chemistry, Department of Chemical Sciences, Faculty of Pharmacy, University of Porto, 4050-313 Porto, Portugal; 4 National Institute of Legal Medicine and Forensic Sciences, I.P., Centre Branch, 3000-548 Coimbra, Portugal; 5 Faculty of Sciences, Lisbon University, 1749-016 Lisboa, Portugal

**Keywords:** Y chromosome, Y-STR, microsatellites, haplotypes, population genetics, genetic diversity, forensic sciences, sexual crimes

## Abstract

Background/Objectives: The Y chromosome is a crucial tool in forensic genetics due to its unique characteristics, such as its haploid inheritance and lack of recombination. Y-STRs (short tandem repeats on the Y chromosome) are widely used for identifying male genetic profiles in DNA mixtures, especially in sexual assault cases where high levels of female DNA hinder autosomal analysis. This study evaluates the applicability of Y-STRs in forensic investigations, addressing their limitations and the impact of advanced technologies, such as rapidly mutating Y-STRs (RM Y-STRs). Methods: A comprehensive literature review was conducted to analyze existing knowledge on the application of Y-STRs in sexual crimes. The study also examines the role of population databases, such as YHRD, in estimating haplotype frequencies and enhancing forensic reliability. Results: Y-STR analysis proves essential for male DNA identification in complex mixtures, with RM Y-STRs enhancing discriminatory power. However, limitations persist, particularly in cases involving closely related male lineages. The population database coverage remains insufficient in regions like Cape Verde, affecting forensic reliability. Case studies demonstrate Y-STR effectiveness in solving cold cases and sexual crimes, reinforcing the need for expanded databases and methodological advancements. Conclusions: Y-STRs play a fundamental role in forensic genetics, particularly in sexual assault investigations. Their integration with advanced sequencing technologies and expanded databases is critical for improving forensic accuracy. Ethical considerations regarding genetic data privacy and potential discrimination must be addressed through clear regulations and forensic best practices.

## 1. Introduction

The analysis of Y-chromosome STRs (Y-STRs) is widely recognized as an essential tool in forensic genetics, particularly in sexual crime cases. These markers are valuable in situations where male DNA is present in minimal quantities or overwhelmed by a large proportion of female DNA. Studies have demonstrated that Y-STRs enable the precise identification of male genetic profiles, even in complex samples, offering a robust approach for forensic investigations [1].

Portugal and Cape Verde were selected as the focus of this study due to their complementary characteristics in terms of population genetics and forensic infrastructure. Portugal has a relatively well-established Y-STR population database; however, despite several completed population studies, the available data remain limited in both geographic coverage and the number of loci analyzed, preventing a comprehensive understanding of the country’s genetic diversity [2,3,4,5,6]. In contrast, Cape Verde faces significant challenges, including limited forensic infrastructure and a small number of haplotypes recorded in international databases such as YHRD [7]. This comparison between two countries allows for an exploration of both opportunities and limitations in distinct forensic contexts, offering insights that may apply to other nations with similar characteristics.

This article examines the forensic applications and implications of Y-STRs, discussing their advantages and limitations, and provides recommendations to maximize their impact on the criminal justice system, with particular emphasis on Cape Verde and Portugal.

## 2. Methods

In November 2024, a comprehensive search was conducted in the PubMed database (U.S. National Library of Medicine) without restrictions on publication date or language to identify the relevant literature on the application of Y-STRs in forensic genetics. The keywords used included “Y-STR”, “Y chromosome”, “forensic genetics”, “DNA mixtures”, “sexual crimes”, “rapidly mutating markers”, and “Y-STR databases.” The review focused on recent advances in Y-STRs applications, their limitations, and their forensic impact in solving complex cases, particularly those involving sexual crimes.

The inclusion criteria comprised peer-reviewed scientific articles that addressed the application of Y-STRs in forensic genetics, particularly in sexual crimes, with emphasis on DNA mixture analysis, population databases, and technological developments such as RM Y-STR markers. Articles discussing relevant ethical, legal, and methodological aspects were also included. Exclusion criteria involved duplicate records, articles not directly related to the forensic application of Y-STRs (e.g., studies with a purely evolutionary or clinical focus), papers without full-text access, and systematic reviews lacking original data or discussion.

Additionally, documents from the National Institute of Statistics of Cape Verde (INE), the Office for Strategy and Studies (GEE), EUROSTAT (the Statistical Office of the European Union), and the United Nations Office on Drugs and Crime (UNODC), as well as the Annual Internal Security Report (RASI), were consulted for complementary data on this topic.

The literature synthesis was performed through a narrative approach, organized around the following thematic axes: (1) characteristics and limitations of Y-STRs; (2) forensic case studies involving Y-STRs; (3) the role of population databases; (4) methodological and technological developments; and (5) ethical implications and public policy recommendations.

## 3. The Y Chromosome and Its Application in Forensic Genetics

The human Y chromosome is a unique structure in the genome, composed of approximately 60 Mb and transmitted exclusively through the paternal lineage. This chromosome, present only in male individuals, is crucial for sex determination, as it is responsible for the development of the testes during embryogenesis. With minimal changes across generations, except for occasional mutations, the Y chromosome has distinct characteristics compared to other human chromosomes, such as haploid inheritance and the absence of recombination (except in two specific regions that pair with the X chromosome). These features have made the Y chromosome an essential tool in forensic genetics, genetic genealogy, and evolutionary research [8,9,10].

## 4. Y-STRs: Exclusive and Highly Informative Markers

Short tandem repeats on the Y chromosome (Y-STRs) are male-specific (XY) markers widely used in forensic analysis due to their specificity and high informativeness. These markers are fundamental for identifying individuals through the paternal lineage, conducting paternity tests, differentiating male relatives, inferring geographical origins, and performing molecular anthropology research [11,12,13].

Due to the absence of recombination on the Y chromosome, Y-STR loci are physically linked, forming haplotypes that are directly transmitted from father to son. This inheritance pattern creates an organized structure of alleles and haplotypes that correlates with geographical and ethnolinguistic patterns, allowing inferences about the population origin of a genetic profile. However, the haploid nature of the Y chromosome presents challenges, as paternally related males often share similar haplotypes, making individualization difficult in forensic investigations [11,14].

Although Y-STR typing allows for the precise identification of male DNA, it presents significant challenges, particularly in the interpretation of both male–male and male–female DNA mixtures, which is especially critical in sexual assault cases with multiple male contributors or samples containing an overwhelming excess of female DNA. The Y chromosome is inherited in a haploid manner and transmitted from father to son, enabling the tracing of paternal lineage through Y-STR analysis. However, the low evidential value of a Y-STR profile, due to the lack of recombination, can be an obstacle in forensic cases, especially in samples with an excess of female DNA. To mitigate this limitation, some studies suggest expanding the set of Y-STR loci with more informative markers. Additionally, while matching a single male Y-STR profile is straightforward, interpreting mixtures requires caution, as coincidental inclusions can occur, as evidenced by a wrongful conviction case based on a coincidental Y-STR inclusion [15].

The use of more sensitive systems and the inclusion of additional markers have proven crucial in increasing accuracy and discrimination in forensic investigations, leading to improved case resolution. Guidelines with recommendations on Y-STR result interpretation in forensic analysis have been established by the DNA Commission of the International Society of Forensic Genetics (ISFG) [14].

## 5. Real Case Studies of Y-STRs in Forensic Investigation

Y-STRs have proven to be essential in various forensic investigations, particularly in cases involving sexual crimes where male DNA is a crucial part of the evidence. Below are two significant cases in which Y-STRs played a central role in solving crimes and identifying suspects.

### 5.1. The Boston Strangler Case

The case of Mary Sullivan, one of the victims of the Boston Strangler, was solved in 2013, nearly 50 years after the crime. Sullivan was murdered in 1964, and the initial investigation failed to resolve the case. After years of speculation, a connection was finally made between the crime and Albert DeSalvo, the man who had confessed to the murders but was never convicted because he had already passed way. The key to solving the case was Y-STR analysis performed on DNA collected from DeSalvo’s nephew. The test indicated a DNA match between the nephew and the sample collected at the crime scene, implicating DeSalvo as Sullivan’s murderer. The exhumation of DeSalvo’s body and subsequent DNA testing confirmed the genetic relationship, establishing his identity as the true perpetrator with a precision of 1 in 220 billion, ruling out other possibilities [16].

### 5.2. The Vaatstra Case in The Netherlands

Another significant case involving Y-STR application occurred in the Netherlands with the case of Marianne Vaatstra, who was brutally raped and murdered in 1999. Despite years of investigation, the case remained unsolved until 2012, when local police implemented an innovative mass DNA collection strategy known as a “DNA dragnet”.

More than 6600 men from the region were invited to provide saliva samples for DNA analysis. Y-STRs were used to identify potential relatives of the perpetrator, as the attacker was unlikely to voluntarily provide a DNA sample. The results revealed a rare Y-STR match in two men from the region, both of whom were ruled out as suspects after autosomal DNA marker analysis. However, the use of Y-STRs allowed investigators to trace the paternal lineage of the perpetrator, ultimately leading to the identification of the culprit. This case demonstrated the value of Y-STRs not only in identifying suspects directly, but also in locating relatives, which enabled the resolution of a long-unsolved case [17,18].

These examples highlight how Y-STRs are essential in investigating cold or archived cases, particularly when DNA mixtures are involved or when the offender’s profile is difficult to access due to the predominant presence of female DNA. The application of Y-STRs not only aids in identifying perpetrators but also helps exclude suspects and strengthen links to the crime scene, offering a powerful tool for investigators. The integration of Y-STRs with other forensic technologies, such as investigative genealogy, has become increasingly useful in resolving old and complex cases [19,20].

## 6. Population Databases: A Critical Necessity

The analysis of Y-STRs has proven to be an essential tool in forensic genetics, particularly in complex cases such as sexual crimes. Despite its limitations, including a reduced exclusion power and the absence of Y-STR profiles in national databases, recent technological advancements and growing scientific recognition have strengthened its use. Several commercially developed kits now offer a greater sensitivity and discriminatory power, making Y-STRs even more effective. However, to optimize their impact on criminal investigations, it is crucial to expand population databases and conduct more extensive studies with real samples, ensuring a more consistent and reliable application in diverse forensic contexts [21].

To maximize the potential of Y-STRs, it is essential to have broad and representative population databases, such as the YHRD (Y-Chromosome Haplotype Reference Database) [22]. These databases allow for the estimation of haplotype frequencies and the quantitative analysis of the probative weight of a Y-STR match. They also consider population substructure, which reflects genetic variations associated with regional, cultural, and ethnolinguistic factors. Statistical models, such as Laplace parameters, are often applied to interpret Y-STR profiles in relation to the populations of origin, thus facilitating the statistical interpretation of DNA evidence [14,23,24,25].

The use of these databases is indispensable, especially in judicial investigations, where the robustness and accuracy of genetic analyses can significantly impact case outcomes [12].

Although countries like the United States, China, and South Korea have thousands of recorded haplotypes for 27 Y-STR loci in YHRD, Cape Verde currently has only 117 haplotypes registered for 17 loci and none for 26 or more loci (https://yhrd.org (accessed on 20 November 2024)), greatly limiting the discriminatory power of forensic analysis and complicating the exclusion or accurate identification of suspects. In comparison, Portugal has haplotypes recorded for 23 Y-STR loci (248 haplotypes), which, although representing progress, remain relatively insufficient when considering the genetic diversity of the Portuguese population. This highlights the urgent need to expand and update these population databases, particularly in underrepresented regions, to strengthen the reliability and probative value of Y-STR analyses in forensic casework [22].

## 7. Forensic Applications and the Investigation of Sexual Crimes

The success of solving forensic cases is directly related to the efficient recovery of DNA at the crime scene and the application of a precise and discriminatory method for genetic analysis [26].

Y-STRs are particularly effective tools in the investigation of sexual crimes due to their male specificity, allowing the exclusion of female DNA contribution and facilitating the analysis of male DNA even when present in very low proportions (<5%) [17,27,28,29,30]. This approach is especially advantageous for interpreting mixed DNA profiles, providing clearer and more accurate conclusions compared to autosomal profiles that include DNA from both sexes [29,31]. Additionally, the use of Y-STRs can be useful for determining the number of contributors in mixtures containing multiple male donors [21].

With advances in forensic DNA analysis, which increasingly employs more sensitive and effective methods, there has been a significant rise in the collection and processing of trace DNA in investigations. These traces are characterized by their unknown origin in bodily fluids or tissues and have become widely analyzed due to improvements in available techniques [32].

Recent studies highlight the need to understand the patterns of male DNA prevalence and transfer, such as in female underwear, to differentiate background traces from crime-related events. Several studies have confirmed that male DNA levels in female underwear are low unless the woman cohabits with a man. Additionally, a consistent trend has been observed in which the amount of male DNA recovered from women’s clothing decreases as the intensity and frequency of physical contact with men decline [32,33,34].

Studies have shown that even 24 to 46 h after a sexual assault, viable Y-STR profiles can still be obtained, as spermatozoa may persist in the vaginal canal for up to three days [35]. Nevertheless, the detection of male DNA can be hindered by the preferential amplification of the female component, requiring complementary methods, such as Y-STR typing, to identify the perpetrator [29,30].

Y-STRs have been widely used in various forensic scenarios, including the exclusion of male suspects, the identification of the paternal lineage of perpetrators, the determination of multiple male contributors in complex biological mixtures, and the inference of population origins of unknown suspects [17].

Most forensic samples obtained in cases of sexual assault are intimate swabs collected from the genital tract of a female victim to identify the DNA of a male perpetrator [36]. However, despite advances in semen detection and recovery, the majority of alleged sexual crimes involving vaginal penetration do not present traces of semen [37].

In situations where there is no presence of semen or spermatozoa (such as in cases of digital penetration, penile penetration without ejaculation, or azoospermic perpetrators, including infertile or vasectomized men), it is known that male epithelial cells from fingers or the penis can be transferred to the vagina and subsequently identified [36,37,38,39].

However, due to the large amount of DNA from female epithelial cells in these samples, identifying the perpetrator’s DNA using autosomal analysis methods becomes extremely challenging. In this context, the analysis of Y-chromosome short tandem repeats (Y-STRs), which are specific to male DNA, has become an essential tool in forensic investigations of sexual assault cases. This method allows the recovery of male DNA profiles even when female DNA is present in amounts exceeding 1000 times [36,40,41].

## 8. Recent Advances: Rapidly Mutating Y-STRs (RM Y-STRs)

The development of rapidly mutating Y-STRs (RM Y-STRs) has marked a significant milestone in forensic genetics. These markers, identified by Ballantyne et al. [42], exhibit mutation rates higher than 1 × 10^−2^ per locus per generation, which allows for a greater discrimination between related individuals, including first-degree relatives. This advancement has significantly enhanced forensic analysis in cases where traditional Y-STR profiles are not sufficiently discriminatory. RM Y-STRs have also facilitated the differentiation of profiles in complex biological mixtures and improved efficiency in solving criminal cases, emerging as one of the most promising innovations in the field [43,44].

## 9. Other Relevant Applications

Beyond their forensic applications, Y-STRs are widely used in historical and genealogical studies. These markers enable the tracing of ancestral human lineages and the inference of migratory routes, providing insights into the genetic history of human populations. They are also valuable in the identification of missing persons and disaster victims, particularly in cases where autosomal DNA does not provide sufficient information [8,17].

## 10. Statistics

### 10.1. Statistics on Cape Verde in Portugal

In 2021, there were 34,093 Cape Verdeans residing in Portugal, representing 4.88% of the immigrant population in the country. Currently, the Cape Verdean population is the third-largest foreign community in Portugal, with the highest concentrations in Lisbon (21,141), Setúbal (6373), and Faro (1896) [45].

### 10.2. Statistics on Cape Verde

According to the National Institute of Statistics of Cape Verde, sexual crimes are among the least reported types of crime. In 2015, 104 cases of sexual abuse of children and minors were recorded, representing 0.4% of all incidents that year, marking an 8.3% increase compared to the previous year (2014). Between 2010 and 2015, there was an average annual increase of 9.9% in the number of reported cases of sexual abuse of children and minors. In this category of crime, female children and minors are the primary victims, accounting for 92.5% of the cases. On the other hand, male individuals aged between 22 and 30 years are the main perpetrators of these crimes. In 2015, 106 cases of sexual assault were also recorded. Compared to the previous year, there was a 15.2% increase in sexual assault incidents. Similarly to child sexual abuse cases, female individuals represent the majority of victims (90.2%), while male individuals are the primary perpetrators of this type of crime [46].

### 10.3. Statistics on Portugal

According to the 2023 Annual Internal Security Report (RASI), the victims of crimes against sexual freedom and self-determination are predominantly female (77.3%), while the defendants are primarily male (94.3%). Among the investigations initiated, the most common type of crime was the sexual abuse of children (39.5%), followed by rape and child pornography, with 20.2% and 12.8%, respectively. More specifically, in rape cases, it was found that 100% of the defendants are male, and 90.7% of the victims are female [47].

### 10.4. Statistics of the European Union

According to the UNODC (United Nations Office on Drugs and Crime) and EUROSTAT (European Union Statistical Office), between 2008 and 2015, rape crimes showed a 47% increase, with the majority of victims being women (85.8%). On the other hand, the suspects (96.5%) and defendants (98.3% of the convicted) are predominantly male [48]. In the EU, according to Eurostat, 231,456 sexual violence offenses were recorded by the police in 2022, representing a 10.3% increase compared to the previous year. This trend has been rising since 2015, except in 2020 [49].

## 11. Implications and Public Policy Recommendations

Although Y-STRs offer powerful tools for forensic investigations, their application raises ethical and legal challenges that cannot be overlooked. The creation and use of genetic databases require a careful balance between investigative benefits and the protection of fundamental rights, such as privacy and non-discrimination. Studies have already shown that the use of these databases can generate concerns about misuse, especially in legal systems where regulatory frameworks are weak or outdated [14,17,50]. Recent studies highlight advancements in technologies, such as the use of next-generation sequencing (NGS), which has increased the accuracy of genetic analyses but also amplified concerns about privacy, particularly in countries where specific guidelines for handling genetic information are still lacking [51]. Ethical issues include, for example, the privacy of genetic data, as the inclusion of profiles in databases, especially of individuals who have not been convicted, may violate basic rights [17,52]. Furthermore, the analysis of Y-STRs, being associated with paternal lineage, may reinforce stereotypes or lead investigations unfairly against specific ethnic or cultural groups, especially in minority populations [23,53]. Another relevant ethical concern is informed consent, as the collection of genetic samples is not always accompanied by clear explanations of their use and implications [40,54]. To maximize the benefits and minimize the risks associated with the use of Y-STRs, it is essential for governments and institutions to adopt comprehensive and specific policies. Firstly, the creation of legislation to regulate the use of genetic databases is needed, defining clear criteria for profile inclusion, privacy protection, and measures against discrimination [17,50]. At the same time, expanding population databases is crucial, especially in countries like Cape Verde, which still have a limited number of registered profiles. This effort can be enhanced through international partnerships with platforms such as the Y-Chromosome Haplotype Reference Database (YHRD) [23,55]. Additionally, specialized training for forensic and judicial professionals is essential to ensure the correct interpretation of genetic data, especially in cases involving complex mixtures or where genetic evidence is the primary proof [14,26,56]. Comparing the use of Y-STRs in Cape Verde and Portugal with countries that have more advanced systems, such as South Korea and China, can provide valuable insights. These nations have developed robust and well-structured databases, covering more than 27 loci, which enables a more precise analysis and greater discriminatory power in investigations. These examples demonstrate how the combination of technological advancements and stringent policies can maximize the positive impact of genetic tools [44,57]. Based on this evidence, it is possible to conclude that Y-STRs have the potential to transform forensic investigations, particularly in cases of sexual crimes. However, for their use to be fully effective, continuous investment in technology, the expansion of population databases, and the adaptation of legal systems to handle genetic evidence in an ethical and reliable manner are necessary. Moreover, collaborative initiatives between countries can ensure a more equitable representation of populations and the continuous scientific validation of the methodologies used. These efforts will not only strengthen justice systems in Cape Verde and Portugal but also serve as a model for other nations facing similar challenges in the use of genetic technologies in criminal investigations.

## 12. Discussion

This study presented highlights the importance of the Y chromosome and Y-STRs in forensic genetics, especially in complex cases of sexual crimes. The ability to identify male DNA in mixtures containing large quantities of female genetic material represents a significant scientific advance. However, despite the well-established applications of this method, it is essential to critically assess the technical and contextual limitations that may affect the reliability of the results.

Among the technical challenges, allelic dropout is a prominent issue, especially in degraded or low-template samples. This phenomenon compromises the completeness of genetic profiles, directly impacting the capacity for comparison and individual identification. Furthermore, DNA degradation (frequently observed in samples exposed to adverse environmental conditions), may impair the uniform amplification of all loci, resulting in partial profiles and limiting the scope of forensic interpretation.

The high sensitivity of amplification protocols employed in Y-STR typing, while enabling the analysis of extremely small quantities of genetic material, also heightens the risk of contamination. This necessitates rigorous laboratory protocols and stringent quality control measures during both the collection and analysis phases.

Another critical consideration involves the representativeness of population databases, such as the Y Chromosome Haplotype Reference Database (YHRD), which are fundamental for estimating the probative value of a genetic profile. Underrepresented populations (such as Cape Verde) may introduce sampling bias and compromise the statistical robustness of the analyses, ultimately limiting the evidential value in forensic casework and increasing the risk of misinterpretation, particularly when inferring geographic origin.

Beyond these technical limitations, it is also essential to consider the ethical and legal implications associated with the use and expansion of genetic databases. In Portugal, Law No. 5/2008, of 12 February establishes clear guidelines for the collection, storage, use, and deletion of DNA profiles, aiming to balance the needs of criminal investigations with the protection of fundamental rights, particularly regarding privacy and informed consent.

In Cape Verde, personal data protection is regulated by Decree-Law No. 13/2007, of 26 February, which classifies genetic data as “sensitive data” and requires explicit consent for its processing. However, to date, no specific regulation has been enacted to address the unique challenges inherent to the forensic use of genetic profiles, leaving significant gaps in the protection of citizens’ rights and in the establishment of best practices for forensic applications.

## 13. Future Perspectives

The progress in the identification of RM Y-STRs represents a significant advancement in the field of forensic genetics, enabling a more precise discrimination between individuals belonging to the same paternal lineage and substantially expanding the potential for solving complex cases. However, the successful implementation of these advances requires not only modernized laboratory infrastructure and validated methodologies but also the continuous training and qualification of forensic experts to ensure the accurate interpretation and application of genetic data.

Simultaneously, the expansion of genetic databases and the use of mass screening strategies, such as those employed in large-scale investigations (e.g., DNA dragnets), must be approached with extreme caution, particularly regarding informed consent, the risk of unauthorized secondary use of genetic data, and the potential stigmatization of minority groups.

The increasing adoption of Y-STR analysis and the enhancement of genetic databases should not be regarded as a standalone solution, but rather as a complementary tool, achieving its full potential only when integrated into a judicial system that is ethical, technically proficient, and socially responsible.

In this context, international cooperation plays a vital role, facilitating not only the exchange of best practices in both laboratory and judicial domains but also the harmonization of data protection standards and ethical procedures, with special consideration given to the national contexts of Portugal and Cape Verde.

The future of forensic genetics and of Y-STR analysis will inevitably depend on maintaining a careful balance between technological innovation, scientific rigor, and the protection of fundamental rights. This balance is essential not only to ensure technical reliability but also to strengthen public trust and uphold the legitimacy of using genetic data in judicial processes.

## 14. Conclusions

The expansion and diversification of Y-STR databases, combined with investments in technology and professional training, are essential to maximize the potential of these tools. At the same time, implementing clear and transparent ethical regulations will be indispensable to ensure public trust in the use of these technologies. By combining these efforts, it will be possible to strengthen forensic investigations and offer more robust and fair judicial responses, both in Cape Verde and Portugal.

The Y chromosome and Y-STRs emerge as indispensable tools in forensic genetics, offering precision in identifying male individuals and inferring paternal lineages. Their application is especially relevant in sexual crime cases, where the unique characteristics of Y-STRs enable effective analysis even in situations involving complex DNA mixtures. Additionally, recent advancements, such as RM Y-STRs, have enhanced their discriminatory capacity, enabling differentiation between individuals with genetic connections and significantly increasing the scope of forensic investigations.

The statistics from Cape Verde and Portugal clearly illustrate the urgency of effective strategies to address sexual crimes. In Cape Verde, the steady increase in reported cases, especially child sexual abuse, reflects a severe social issue, with most victims being young women and the perpetrators adult men. In Portugal, the prevalence of sexual crimes such as child abuse and rape, with nearly all suspects being male, reinforces the need for robust genetic identification methods. These data, along with European trends showing a rise in sexual violence, underline the importance of tools such as Y-STRs in supporting criminal justice.

Therefore, the use of Y-STRs, combined with extensive and representative population databases, emerges as a viable and effective solution to assist forensic investigations, especially in contexts where conventional methods face limitations. By integrating advanced technologies with a detailed understanding of regional realities, as demonstrated by the cases in Cape Verde and Portugal, it is possible to significantly improve the response to sexual crimes and strengthen the justice system.

While this article has focused on the specific realities of Cape Verde and Portugal, its findings can be extended to other countries that share similar forensic and infrastructural challenges. These insights may contribute to the development of forensic strategies tailored to local contexts while ensuring the protection of individual rights and ethical standards in the handling of genetic data.

Furthermore, investigations should be accompanied by clear regulations on the ethical use of genetic information, ensuring privacy and non-discrimination. The future of forensic investigations will depend on the combination of these advanced technologies with the continuous education of justice professionals and the implementation of robust public policies that ensure efficiency and equity across legal systems.

## Data Availability

No new data were created or analyzed in this study.

## Abbreviations

The following abbreviations are used in this manuscript:

DNA	Deoxyribonucleic Acid
EUROSTAT	Statistical Office of the European Union
INE	National Institute of Statistics
ISFG	International Society of Forensic Genetics
NGS	Next-Generation Sequencing
RASI	Annual Internal Security Report
RM Y-STR	Rapid Mutation Y-Chromosome Short Tandem Repeats
UNODC	United Nations Office on Drugs and Crime
Y-STR	Short Tandem Repeats of the Y Chromosome
